# Social patterning in grip strength and in its association with age; a cross sectional analysis using the UK Household Longitudinal Study (UKHLS)

**DOI:** 10.1186/s12889-018-5316-x

**Published:** 2018-03-21

**Authors:** Caroline Carney, Michaela Benzeval

**Affiliations:** 1 Department of Health Policy, Cowdray House, London School of Economics and Political Science, Houghton Street, London, WC2A 2AE UK; 2 Institute for Social and Economic Research (ISER), University of Essex, Wivenhoe Park, Colchester, CO4 3SQ UK

**Keywords:** Grip strength, Life-course, Socio-economic position, Age, UKHLS, Cross-sectional

## Abstract

**Background:**

Grip strength in early adulthood and midlife is an important predictor of disability, morbidity and mortality in later life. Understanding social patterning in grip strength at different life stages could improve insight into inequalities in age-related decline and when in the life course interventions could prevent the emergence of inequalities.

**Methods:**

Using United Kingdom Household Longitudinal Study (UKHLS) data on 19,292 people aged 16 to 99, fractional polynomial models were fitted to identify which function of age best described its association with grip strength. Linear regressions were used to establish whether socio-economic position (SEP), as measured by maternal education, highest educational qualification and income, was associated with grip strength. To test whether the association between age and grip strength was modified by SEP, interactions between SEP and the age terms were added. Differentiation was used to identify the age at which grip strength was highest for men and women and predicted levels of grip strength at peak were compared.

**Results:**

SEP is significantly associated with grip strength on all SEP measures, except education for men. Grip strength is highest at a younger age, and less strong for all measures of disadvantage for women and most measures for men. Interaction terms were not statistically significant indicating that the association between age and grip strength was not modified by SEP. Grip strength peak was 29.3 kg at age 33 for women with disadvantaged childhood SEP compared with 30.2 kg at age 35 for women with advantaged childhood SEP.

**Conclusion:**

The SEP differences in age and level of peak grip strength could be indicative of decline in muscle strength beginning earlier and from a lower base for disadvantaged groups. This could impact on the capacity for healthy ageing for those with disadvantaged SEP.

**Electronic supplementary material:**

The online version of this article (10.1186/s12889-018-5316-x) contains supplementary material, which is available to authorized users.

## Background

Grip strength is a measure of upper body strength that is indicative of overall muscle function. Grip strength is known to increase in early adulthood and decline from midlife onwards [1]. Studies where grip strength was measured in early adulthood and midlife have found that it is predictive of disability, morbidity and all cause and cardiovascular mortality in later life [2–6]. Grip strength was associated with more ageing markers than chronological age in research using the Hertfordshire Cohort Study [7]. Research into the social determinants of grip strength has had contradictory results with some research finding evidence of social patterning [8–14] while other research did not [15–18].

Understanding the social patterning of grip strength therefore could improve insights into how to reduce poor health at older ages. It is hypothesised that socio-economic position (SEP) is associated with grip strength and affects the age and level at which it peaks due to socially patterned development in utero, growth in childhood and later adult health behaviours. It is posited here that a material lack of resources influences mother’s diets which in turn influences the number of muscle fibres and muscle quality set in utero [19, 20]. Growth in childhood and adolescence is known to be associated with grip strength [21] and is hypothesised to be socially patterned likewise through material resources and diet. Physical activity in adulthood has been associated with grip strength [15, 22–24]. As physical activity is socially patterned [25], it is proposed here that grip strength is affected by adult SEP.

All previous research on SEP’s association with grip strength has been based on cohort studies where the sample is a similar age or within a particular range. This limits understanding of social patterning in grip strength at different life stages. Exploration of grip strength in a sample with a wide age range has been undertaken in previous research [1] but without consideration of social patterning. There is no research thus far exploring social patterning in grip strength across all ages or identifying when inequalities emerge. The objective of this paper is to investigate whether SEP is associated with grip strength in a full adult age range and to identify at what age grip strength is highest and whether this age and level of strength vary on the basis of SEP.

## Methods

### Study sample

Data are from participants in the Nurse Health Assessment (NHA) [26] of Understanding Society: the UK Household Longitudinal Study (UKHLS) [27]. British residents who were members of the UKHLS General Population Sample (GPS) in wave two and members of the British Household Panel Survey (BHPS) sample in wave three were invited to participate in the NHA.

The GPS is a stratified, clustered, equal probability sample of residential addresses drawn to a uniform design throughout the whole of the UK [28] and the BHPS began in 1991 as a stratified random sample [29]. The NHA interviews were conducted approximately 5 months after participation in the main interview, beginning in May 2010. A trained nurse visited respondent’s homes and collected a number of measures of physical functioning, anthropometric measures and blood samples.

There were 43,747 adult, British resident members of the GPS and BHPS who gave a full interview in waves two and three respectively. Eligibility was restricted to those who interviewed in English, were not pregnant and in the second year of wave two, selection into the NHA was further restricted to 81% of the primary sampling units in England for the GPS component. There were 35,613 eligible to participate in the NHA and 20,644 took part [30]. This was a response rate of 58.6% from the GPS and 56.7% from the BHPS.

### Measurement

Grip strength was measured with a Smedley Dynometer. Respondents stood up with their arms by their sides and holding the dynometer with the dial facing outward, they took three measurements with each hand. Grip strength is measured in kilograms (kg) and the greatest grip strength measurement obtained from the dominant hand is used [30]. Respondents were ineligible to provide a grip strength measurement if they had a hand injury or pain. There were 19,292 respondents who provided a grip strength measurement, 6.6% of the NHA sample were ineligible. Respondents who reported a grip strength of zero were investigated as this may be considered an implausible measurement. Of the eight in total who reported this, all reported either a limiting disability or illness or were elderly. Thus these were considered plausible results and retained in the analysis.

Maternal education was used to capture SEP in the in utero and childhood period. Previous research has shown that maternal education is associated with nutrition and health behaviours in pregnant women and with birth weight [31–33] as well as with childhood diet [34]. Respondents were asked whether their mother had gone to school at all, left school with no qualifications, left school with some qualifications, gained higher qualification, or gained a degree or higher degree; responses of ‘do not know’ and ‘other’ were reclassified as missing. The response categories were collapsed: ‘no school or qualifications’ indicating disadvantaged SEP and ‘some qualifications, post school qualifications or degree or higher’ indicating advantaged SEP. Maternal education was collected across waves one and two for GPS respondents and a proportion of BHPS respondents provided this in wave 13 of BHPS. Responses were combined into one maternal education variable.

The measures employed for adult SEP were educational attainment and income. Much previous research on grip strength has used occupation to measure SEP [8–11, 13, 15–18]. However it was hypothesised here that the potentially beneficial effect of physical activity on grip strength via manual occupation could prevent identification of the adverse impact of disadvantaged SEP. Education and income were collected in wave two of UKHLS. There was approximately a 1 year lag between collection of these and grip strength measurement for the BHPS sample compared to 5 months for the GPS.

For educational attainment, the derived measure of highest qualification was used which is categorised into degree, other higher qualification, A level, GCSE, other qualification and no qualification. These were grouped into ‘A level and higher’ and ‘GCSE and lower’.

Income data were collected from all household members and used to construct net household income in the month before interview. There was high non-response to some components of income [35], the derived measure of household income includes imputed income data reducing missing data to 0.5% in this analytical sample. Household income was equivalised for household size using the modified OECD equivalence scale. Income was categorised into quintiles and a binary measure of whether respondents were in the lowest income quintile or not was used.

Age was calculated based on date of interview and date of birth, then confirmed by the nurse during the interview.

Height is sometimes adjusted for in studies on grip strength as body size is a determinant of grip strength. However, height may act as a marker for SEP as well as hereditable traits. It was excluded from the analysis as it would attenuate the association between SEP and grip strength rather than elucidate it.

### Statistical methods

Investigating whether grip strength’s association with age varied by SEP required specifying the shape of the association between grip strength and age. Fractional polynomials were used to assess which function of age best described the association. As this required reducing the simplicity of the models, Aikake’s Information Criterion (AIC) was used to test whether these functions of age fitted the data better than simple quadratic functions [36]. Quadratic functions were used for comparison to the other functional forms as initial inspection using scatter plots indicated the association may be quadratic.

Linear regressions were used to establish whether each SEP measure was associated with grip strength with adjustment for the age terms specified by the fractional polynomial.

To identify the age of highest grip strength for advantaged and disadvantaged SEP groups, linear regressions between age and grip strength were stratified by advantaged and disadvantaged SEP for each measure. The fractional polynomial function was then differentiated to find the age of highest grip strength for each SEP group.

To compare the levels of predicted grip strength at the age it is highest and to identify whether SEP modified the age association, age terms were centred to the age of peak grip strength for advantaged and disadvantaged groups and an interaction with the SEP term was included. This was undertaken for each SEP measure.

The analysis was stratified by gender due to gender differences in grip strength. Available-case analysis is used to retain sample size and the number of observations varies across models, thus the models are not comparable. The cross sectional weight for the combined NHA sample is used to adjust for unequal selection probabilities and differential nonresponse to the NHA. Analysis was undertaken using Stata 14 [37].

As a sensitivity check, models were rerun with adjustment for height to assess if this negated the SEP associations. Due to data on SEP being collected one wave prior to the NHA for the BHPS sample and in the same wave for the GPS, analysis was rerun including a control variable based on sample membership. Adjustment for height or for sample membership did not substantially change the results. Analyses were rerun substituting ‘no qualifications’ for ‘GCSE or lower qualification’ to measure low education and using the two lowest income quintiles to indicate low income. A similar check was not conducted with maternal education due to categories of qualification for this measure being derived in a different way. The associations found were consistent, being more pronounced for education and less pronounced for income. A sensitivity check was conducted substituting the fractional polynomial terms for the inclusion of a quadratic term. This did not alter the associations found between SEP and grip strength but did alter findings in the social patterning at the age and level of highest grip strength, social patterning became more pronounced.

## Results

### Descriptive data

Table 1 describes the characteristics of grip strength respondents. Response to grip strength was 94.4%. The profile of non-respondents was compared to those who responded. Non-respondents were more likely to be older, female, have a long-term illness or disability, lower education and poorer health behaviours than the NHA sample. This suggests that there has been exclusion from the analytical sample of those who are most disadvantaged and who have poorest health, which limits the generalisability of the findings. Mean age was 47.7 years (standard deviation (SD) 18.6) and 55% were female. There were 39.9% of respondents who reported no maternal qualifications and 49.8% reported their own educational level as GCSE or lower. Missing data are low on each measure aside from maternal education, where it is 13.9%. Non-responders to maternal education were more likely to be male, younger, have disadvantaged adult SEP and poorer health behaviours. This indicates exclusion of those who are disadvantaged, again limiting the findings generalisability.

**Table 1:** Descriptive characteristics for grip strength respondents

Grip strength respondents	
	*N* = 19,292
Age	
Mean (SD)	47.7 (18.6)
Gender	
Female	55.0%
Childhood SEP	
Maternal education	
Some qualifications or higher	46.2%
No qualifications	39.9%
Missing	13.9%
Adult SEP	
Education	
A level or higher	49.0%
GCSE or lower	49.8%
Missing	1.3%
Income	
Lowest quintile	20.8%
Missing	0.5%

Women had a mean grip strength of 26.7 kg (SD 7.1), ranging between 0 kg and 55 kg. For men, the mean grip strength was 42.8 kg (SD 10.3) ranging from 0 kg to 80 kg.

### Association between age and grip strength

Using a fractional polynomial to assess the shape of the association between age and grip strength indicated that age to the power of 0.5 and age in its linear form was the best fit for women; and that age to the power of − 1 and age to the power of 2 gave the best fit for men. As this required reducing the simplicity of the models, AIC was used to examine whether using the functions of age suggested by the fractional polynomial was the best approach compared to quadratic age. A smaller AIC indicates a better fitting model. In each case the model suggested by the fractional polynomial had a marginally smaller AIC. In order to compare the AIC statistics an approach suggested by Burnham & Anderson [36] was used to test the probability that the model with a smaller AIC was significantly better (see Additional file 1 for results). For both men and women, the terms produced by the fractional polynomial models were a significantly better fit and are employed throughout the rest of the paper.

### Association between SEP and grip strength

Table 2 shows the association between each SEP measure and grip strength. Women reporting no maternal qualifications had grip strength approximately one kilogram (confidence interval (CI) -1.398 kg to − 0.753 kg) weaker than those with higher maternal education, as did those reporting their education being up to GCSE education in comparison to those with higher education. Those in the lowest income quintile had grip strength 0.461 kg (− 0.809 kg to − 0.114 kg) weaker than those in higher quintiles. Men reporting no maternal qualifications had grip strength approximately one kilogram lower than those with higher maternal education. No significant association was found with own education. Men in the lowest quintile had grip strength 1.590 kg (− 2.201 kg to − 0.990 kg) weaker than men with higher incomes.

**Table 2:** Association^a^ between SEP measures and grip strength for women and men

	Value	Coefficient (95% CI)	*p*
Women			
Maternal education (*N* = 8576)	Some qualifications or higher as reference		
	No qualifications	−1.075 (− 1.398 to −0.753)	<.001
Education (*N* = 9824)	A level or higher as reference		
	GCSE or lower	−1.034 (− 1.320 to −0.747)	<.001
Income (*N* = 9848)	All other quintiles as reference		
	Lowest quintile	−0.461 (− 0.809 to − 0.114)	<.05
Men			
Maternal education (*N* = 6677)	Some qualifications or higher as reference		
	No qualifications	−1.118 (−1.660 to −0.576)	<.001
Education (*N* = 7799)	A level or higher as reference		
	GCSE or lower	0.115 (−0.347 to 0.578)	.625
Income (*N* = 7811)	All other quintiles as reference		
	Lowest quintile	−1.590 (−2.201 to −0.990)	<.001

^a^Adjusted for age terms

### Social patterning in the age of highest grip strength

Table 3 shows the age at which grip strength is highest for men and women by each SEP measure (see Additional file 2 for regressions used to produce these results). On average, grip strength is highest at age 34 for women and at age 36 for men. Grip strength is strongest at age 33 for women reporting no maternal qualifications compared to 35 for those reporting higher maternal education. Women with their own education up to GCSE level achieve strongest grip strength 2.2 years younger than those with higher education. For income, women in the lowest quintile have highest grip strength 2.4 years earlier than those in higher quintiles. Conversely, men reporting no maternal qualifications had their highest grip strength approximately half a year older than those with higher maternal education. However, men reporting GCSE or lower levels of education have their highest grip strength 2 years earlier than those with higher levels of education. There was no difference in the age of highest grip strength by income for men.

**Table 3:** Age at highest grip strength by SEP in women and men

	Age at highest grip strength	
SEP measure	Advantaged SEP	Disadvantaged SEP
Women		
Childhood SEP	35.3 years	33.3 years
Adult SEP		
Education	35.4 years	33.2 years
Income	34.5 years	32.1 years
Men		
Childhood SEP	36.8 years	37.4 years
Adult SEP		
Education	37.5 years	35.7 years
Income	36.7 years	36.7 years

### Social patterning in the level of highest grip strength

Table 4 shows the level of grip strength achieved at the age of highest grip strength by SEP (see Additional file 3 for regressions used to produce these results). On average, predicted grip strength at peak is 29.8 kg (29.6 kg to 30.0 kg) and 47.6 kg (47.3 kg to 47.9 kg) for women and men respectively. Women reporting no maternal qualifications had a highest grip strength of 29.3 kg (28.9 kg to 29.7 kg) compared to 30.2 kg (29.9 kg to 30.5 kg) for those reporting higher levels of maternal education. Those with GCSE or lower education had a peak grip strength 0.7 kg weaker than those with higher education while those in the lowest income quintile had predicted grip strength only marginally weaker than higher income counterparts. Grip strength was 1.2 kg weaker at peak for men reporting no maternal qualifications compared to those reporting higher maternal education. Men with GCSE or lower education had grip strength 0.8 kg stronger at peak than those with higher education. Men in the lowest income quintile had a peak predicted grip strength approximately two kilograms weaker than men with higher income.

**Table 4:** Level of highest grip strength by SEP in women and men

SEP measure	Advantaged SEP	Disadvantaged SEP
Women		
	Predicted grip strength at peak (95% CI)	Predicted grip strength at peak (95% CI)
Childhood SEP		
Maternal education	30.2 kg (29.9 kg to 30.5 kg)	29.3 kg (28.9 kg to 29.7 kg)
Adult SEP		
Education	30.1 kg (29.8 kg to 30.3 kg)	29.4 kg (29.1 kg to 29.7 kg)
Income	29.9 kg (29.7 g to 30.1 kg)	29.5 kg (29.1 kg to 30.0 kg)
Men		
	Predicted grip strength at peak (95% CI)	Predicted grip strength at peak (95% CI)
Childhood SEP		
Maternal education	48.0 kg (47.5 kg to 48.5 kg)	46.8 kg (46.2 kg to 47.4 kg)
Adult SEP		
Education	47.2 kg (46.8 kg to 47.7 kg)	48.0 kg (47.5 kg to 48.5 kg)
Income	47.9 kg (47.6 kg to 48.3 kg)	45.9 kg (44.9 kg to 46.7 kg)

**Fig. 1:**
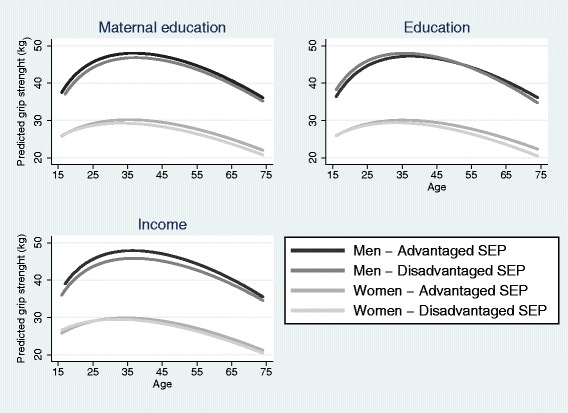
Trajectory of grip strength for men and women by disadvantaged or advantaged SEP. Predicted probabilities from data up to age 75 for men and women obtained from regressing fractional polynomial age terms on grip strength stratified by disadvantaged and advantaged SEP on maternal education, own education and income

The interaction terms in these regressions were not significant indicating that SEP did not significantly modify the associations between age and grip strength. Figure 1 shows the predicted probabilities for grip strength up to age 75 for men and women stratified by disadvantaged and advantaged SEP on each measure. Though the interaction terms were not significant, there is a near consistent pattern of lower grip strength for disadvantaged SEP groups, particularly after it peaks. For maternal education, the difference in grip strength is consistent for men across the lifespan, among women the trajectories diverge after early adulthood. Men educated up to GCSE have stronger predicted grip strength in early adulthood but the difference inverts in later life. The differences based on education become steeper across the life course for women. Grip strength peaks at a weaker level for men in the lowest income quintile, the trajectories converge somewhat in later life. Despite stronger grip strength at very early adulthood, there is less growth and a longer decline for women in low income.

## Discussion

### Key results

On average, grip strength is highest at age 34 for women and at age 36 for men, with predicted highest measurements of 29.8 kg (29.6 kg to 30.0 kg) and 47.6 kg (47.3 kg to 47.9 kg) respectively. Low maternal education and being in the lowest income quintile were significantly associated with weaker grip strength. Own education was associated with grip strength for women but not men. Women with disadvantaged SEP had a highest grip strength approximately 2 years younger and at a weaker level than women with advantaged SEP on each measure. This indicates grip strength decline may begin earlier and from a lower peak. The pattern of results across SEP measures was less consistent for men. Grip strength was strongest at a younger age for men reporting disadvantage on maternal education but the converse was found on the basis of own education. Grip strength’s peak was at a lower level for men reporting disadvantage on maternal education but was at a higher level for those reporting disadvantage on own education. For men, the greatest SEP difference in peak grip strength was on the basis of income where it was two kilograms weaker for those in the lowest income quintile.

This research builds upon the findings of other studies on grip strength and SEP by exploring the association in a full age range with SEP measures from different stages of the life course. In contrast to much research in this area [8–11, 13, 15–18] occupation was not used to measure SEP. Many of the studies using occupation did not find a significant association between occupation and grip strength [8–10, 15–18] though some of these found significant associations with other SEP measures [8–10]. Having a manual or physically active occupation may be protective against some of the effects of disadvantage for grip strength. That this study found an association between SEP and grip strength where other studies did not may also be due using a full rather than narrow age range. The narrower SEP differences in grip strength found in men compared to women found here may be explained by men with disadvantaged SEP being more likely to have engaged in manual labour, which could be protective.

Physical activity may mediate the association found here between SEP and grip strength. Physical activity is known to be socially patterned [25] and has been found to be important for grip strength [15, 22–24], however studies reviewed here which considered SEP as well as physical activity did not explore mediation [15, 22]. The gender difference in associations found here may also be influenced by differences in leisure time physical activity, men tend to participate more in sports while walking accounts for a greater proportion of women’s leisure time physical activity [38].

This research found disadvantaged SEP was associated with lower grip strength and found the age and level of peak grip strength was younger and weaker for those with disadvantaged SEP, particularly for women. This may affect the capacity for healthy ageing among those with disadvantaged SEP. No previous research has explored SEP differences in grip strength’s peak and decline however previous research has found that these are important; the rate of decline of grip strength and the level from which it declines were predictive of mortality in men [6]. The SEP differences in grip strength in adulthood may have little immediate implications but could have long-term consequences due to the association of grip strength with healthy ageing, frailty, disability and mortality.

### Limitations

Cross sectional data restricts the assumptions that can be made about grip strength’s growth, peak and decline and it is not possible to differentiate between cohort, period and age effects here. The analysis shows a cross section of grip strength across adulthood rather than the trajectory of individual’s grip strength across their life course.

As maternal education was collected retrospectively, it may be more prone to measurement error though previous research has shown that retrospective recall is effective [39]. This measure was a proxy for environment in utero, this would be better measured by birth weight but it was not available. The relevance of GCSE and lower education to indicate disadvantaged SEP may vary across age groups. Educational attainment has increased over time and what constitutes low education is not consistent across age groups.

The weighted analysis conducted here means the results are representative of the British household population but this did not include those in institutions such as homes for the elderly or hospitals. This excluded those who may have very low grip strength though this limitation would apply to most large-scale collections of grip strength data. Those who are sick and elderly are more likely to have been excluded from grip strength measurement and would be likely to have weak grip strength meaning that associations may be under estimated. Using the NHA weight for the combined GPS and BHPS samples allows the results from this analysis to be generalised to the household population of Britain, however, the generalisability of these results is undermined by the large non-response to maternal education.

## Conclusions

This is a cross sectional descriptive paper on a representative sample of adults of all ages, which is a novel contribution to research on inequalities in grip strength. Understanding when development peaks and how steeply it declines for different groups enables policy makers to identify at which life stage intervention may be useful and whether to focus on ways of enhancing development or slowing decline. It is important to understand this by SEP, so that policymakers are aware of how interventions in this area may affect inequalities. This research suggests that disadvantaged SEP is associated with weaker grip strength and that grip strength among disadvantaged groups peaks at a younger age and a weaker level on all of the SEP measures explored for women and on some of these for men. This has implications for inequalities in healthy ageing. Research into whether the initial development can be improved may identify interventions to reduce such inequalities. Further research into whether physical activity mediates SEP’s association with grip strength could provide insights into whether physical activity can be protective and alleviate decline.

## Additional files

Additional file 1: Comparison of quadratic and fractional polynomial models. (DOCX 19 kb)

Additional file 2: Age terms regressed on grip strength stratified by advantaged or disadvantaged SEP. (DOCX 25 kb)

Additional file 3: Models producing highest predicted grip strength. (DOCX 23 kb)

## Abbreviations

AIC: Aikake’s information criterion
BHPS: British household panel survey
CI: Confidence interval
GPS: General population sample
KG: Kilogram
N: Number of observations
NHA: Nurse health assessment
SD: Standard deviation
SEP: Socio-economic position
UKHLS: United Kingdom Household Longitudinal Study
