# Supplementary material for: Social patterning in grip strength and in its association with age; a cross sectional analysis using the UK Household Longitudinal Study (UKHLS)
Source: BMC Public Health. 2018 Mar 21;18:385. doi: 10.1186/s12889-018-5316-x (PMC5863489; doi:10.1186/s12889-018-5316-x)
Supplement: Supplementary file 1 — Comparison of quadratic and fractional polynomial models. (DOCX 19 kb) [file 12889_2018_5316_MOESM1_ESM.docx]

**Additional file 1: Comparison of quadratic and fractional polynomial models**

To compare AIC statistics; a *p*-value was produced using an approach suggested by Burnham & Anderson, *p*=(exp(AIC_fp_-AIC_quad_))/2), to test whether the model with a smaller AIC was significantly better [[38](#_ENREF_38)].

*Table S1 AIC statistics and comparison between quadratic and fractional polynomials models*

|  | Age terms | AIC | *p* |
| --- | --- | --- | --- |
| Women |  |  |  |
|  | Age & age^2 | 64513.94 |  |
|  | Age^0.5 & age^1 | 64458.85 | <.001 |
|  |  |  |  |
| Men |  |  |  |
|  | Age & age^2 | 57219.6 |  |
|  | Age^-1 & age^2 | 57144.35 | <.001 |
