# Supplementary material for: Social patterning in grip strength and in its association with age; a cross sectional analysis using the UK Household Longitudinal Study (UKHLS)
Source: BMC Public Health. 2018 Mar 21;18:385. doi: 10.1186/s12889-018-5316-x (PMC5863489; doi:10.1186/s12889-018-5316-x)
Supplement: Supplementary file 2 — Age terms regressed on grip strength stratified by advantaged or disadvantaged SEP. (DOCX 25 kb) [file 12889_2018_5316_MOESM2_ESM.docx]

**Additional file 2: Age terms regressed on grip strength stratified by advantaged or disadvantaged SEP**

Supplementary table 2 shows the coefficients produced by regressing age to the power of -0.5 and linear age regressed on grip strength for women. To gain the age of highest grip strength, the formula ${((-0.5\times{\_b}_{1})\div({\_b}_{2}))}^{2}$ was used.

*Table S2 Age terms regressed on grip strength for women*

|  | Value | Coefficient (95% CI) | *p* |
| --- | --- | --- | --- |
| Maternal education |  |  |  |
| No school/qualifications  (N=4,231) |  |  |  |
|  | **Age^-1** | 12.205 (10.476 to 13.933) | <.001 |
|  | **Age^2** | -1.058 (-1.178 to -0.938) | <.001 |
|  | **Constant** | -5.918 (-12.060 to 0.224) | <.001 |
| Some or higher education  (N=4,345) |  |  |  |
|  | **Age^-1** | 13.773 (12.336 to 15.210) | <.001 |
|  | **Age^2** | -1.159 (-1.269 to -1.049) | <.001 |
|  | **Constant** | -10.730 (-15.304 to -6.156) | <.001 |
|  |  |  |  |
| Education |  |  |  |
| GCSE or lower  (N=5,197) |  |  |  |
|  | Age^-1 | 12.679 (11.408 to 13.950) | <.001 |
|  | Age^2 | -1.101 (-1.193 to -1.010) | <.001 |
|  | Constant | -7.069 (-11.357 to -2.781) | <.001 |
| A level or higher  (N=4,059) |  |  |  |
|  | Age^-1 | 13.076 (11.591 to 14.561) | <.001 |
|  | Age^2 | -1.099 (-1.210 to -0.988) | <.001 |
|  | Constant | -8.805 (-13.669 to -3.942) | <.001 |
|  |  |  |  |
| Income |  |  |  |
| Lowest income quintile  (N=2,046) |  |  |  |
|  | Age^-1 | 11.943 (10.030 to 13.856) | <.001 |
|  | Age^2 | -1.054 (-1.192 to -0.915) | <.001 |
|  | Constant | -4.321 (-10.684 to 2.042) | <.001 |
| All other income quintiles  (N=7,502) |  |  |  |
|  | Age^-1 | 12.571 (12.571 to 14.728) | <.001 |
|  | Age^2 | -1.163 (-1.242 to -1.083) | <.001 |
|  | Constant | -10.182 (-13.755 to -6.609) | <.001 |

Table S3 shows the coefficients produced by regressing age to the power of -1 and age to the power of 2 regressed on grip strength for men. To gain the age of highest grip strength, the formula $∛(\frac{{\_b}_{1}}{2\times_{{\_b}_{2}}})$ was used.

*Table S3 Age terms regressed on grip strength for men*

|  | Value | Coefficient (95% CI) | *p* |
| --- | --- | --- | --- |
| Maternal education |  |  |  |
| No school/qualifications  (N=3,321) |  |  |  |
|  | Age^-1 | -453.338 (-529.522 to -377.154) | <.001 |
|  | Age^2 | -0.004 (-0.005 to-0.004) | <.001 |
|  | Constant | 65.007 (62.554 to 67.460) | <.001 |
| Some or higher education  (N=3,356) |  |  |  |
|  | Age^-1 | -432.059 (-478.877 to -385.242) | <.001 |
|  | Age^2 | -0.004 (-0.005 to -0.004) | <.001 |
|  | Constant | 65.633 (62.554 to 67.460) | <.001 |
|  |  |  |  |
| Education |  |  |  |
| GCSE or lower  (N=3,740) |  |  |  |
|  | Age^-1 | -416.245 (-464.130 to -368.361) | <.001 |
|  | Age^2 | -0.004 (-0.005 to -0.004) | <.001 |
|  | Constant | 65.550 (63.697 to 67.403) | <.001 |
| A level or higher  N=4,059) |  |  |  |
|  | Age^-1 | -432.048 (-481.165 to -382.931) | <.001 |
|  | Age^2 | -0.004 (-0.005 to -0.004) | <.001 |
|  | Constant | 64.553 (62.644 to 66.462) | <.001 |
|  |  |  |  |
| Income |  |  |  |
| Lowest income quintile  (N=1,399) |  |  |  |
|  | Age^-1 | -405.475 (-481.571 to -329.378) | <.001 |
|  | Age^2 | -0.004 (-0.005 to -0.004) | <.001 |
|  | Constant | 62.403 (59.312 to 65.494) | <.001 |
| All other income quintiles  (N=6,412) |  |  |  |
|  | Age^-1 | -429.895 (-466.098 to -393.692) | <.001 |
|  | Age^2 | -0.005 (-0.005 to -0.004) | <.001 |
|  | Constant | 65.589 (64.163 to 67.015) | <.001 |
