# Supplementary material for: Social patterning in grip strength and in its association with age; a cross sectional analysis using the UK Household Longitudinal Study (UKHLS)
Source: BMC Public Health. 2018 Mar 21;18:385. doi: 10.1186/s12889-018-5316-x (PMC5863489; doi:10.1186/s12889-018-5316-x)
Supplement: Supplementary file 3 — Models producing highest predicted grip strength. (DOCX 23 kb) [file 12889_2018_5316_MOESM3_ESM.docx]

**Additional file 3: Models producing highest predicted grip strength**

These models were produced by using age terms centred to the age of highest grip strength specific to whether SEP was disadvantaged or advantaged to obtain predicted grip strength at that age. Results for women are shown on Supplementary table 4.

*Table S4 Age terms regressed on grip strength with SEP interaction for women*

| Women | Value | Coefficient (95% CI) | *p* |
| --- | --- | --- | --- |
| Maternal education  (N=8,576) |  |  |  |
|  | Some or more schooling | 30.190 (29.930 to 30.451) | <.001 |
|  | No qualifications | 29.270 (28.866 to 29.673) | <.001 |
|  | Age | -1.159 (-1.269 to -1.049) | <.001 |
|  | Age^.5 | 13.773 (12.336 to 15.210) | <.001 |
|  | Age * no qualifications | 0.101 (-0.062 to 0.263) | .226 |
|  | Age^.5 * no qualifications | -1.568 (-3.816 to 0.679) | .171 |
| Education  (N=9,824) |  |  |  |
|  | A level or higher | 30.081 (29.832 to 30.331) | <.001 |
|  | GCSE or lower | 29.408 (29.101 to 29.715) | <.001 |
|  | Age | -1.102 (-1.193 to -1.010) | <.001 |
|  | Age^.5 | 13.076 (11.591 to 14.561) | <.001 |
|  | Age * GCSE or lower | -0.397 (-2.351 to 1.557) | .690 |
|  | Age ^.5 * GCSE or lower | -0.003 (-0.146 to 0.141) | .972 |
| Income  (N=9,848) |  |  |  |
|  | All other quintiles | 29.872 (29.068 to 29.972) | <.001 |
|  | Lowest quintile | 29.520 (29.658 to 30.085) | <.001 |
|  | Age | -1.163 (-1.242 to -1.083) | <.001 |
|  | Age^.5 | 11.943 (10.031 to 13.855) | <.001 |
|  | Age * lowest quintile | 0.109 (-0.050 to 0.269) | .179 |
|  | Age ^.5 * lowest quintile | -1.707 (-3.901 to 0.488) | .127 |

Table S5 shows estimates from models using age terms centred to the age of highest grip strength specific to whether SEP was disadvantaged or advantaged to gain predicted grip strength at that age for men.

*Table S5 Age terms regressed on grip strength with SEP interactions for men*

|  | Value | Coefficient (95% CI) | *p* |
| --- | --- | --- | --- |
| Maternal education  (N=6,677) |  |  |  |
|  | Some or more schooling | 48.025 (47.544 to 48.506) | <.001 |
|  | No qualifications | 46.825 (46.237 to 47.413) | <.001 |
|  | Age^-1 | -453.338 (-529.507 to-377.168) | <.001 |
|  | Age^2 | -0.004 (-0.005 to -0.004) | <.001 |
|  | Age ^-1* no qualifications | 21.279 (-68.125 to 110.682) | .641 |
|  | Age ^2 * no qualifications | 1.082e-6 (-0.001 to 0.001) | .996 |
| Education  (N=7,799) |  |  |  |
|  | A level or higher | 47.236 (46.790 to 47.681) | <.001 |
|  | GCSE or lower | 48.025 (47.513 to 48.537) | <.001 |
|  | Age^-1 | -416.245 (-464.121to -368.369) | <.001 |
|  | Age^2 | -0.004 (-0.004 to -0.004) | <.001 |
|  | Age^-1 * GCSE or lower | -15.803 (-84.388 to 52.782) | .652 |
|  | Age^2 * GCSE or lower | -0.001 (-0.001 to 9.46e-4) | <.05 |
| Income  (N=7,811) |  |  |  |
|  | All other quintiles | 47.910 (47.546 to 48.274) | <.001 |
|  | Lowest quintile | 45.850 (44.984 to 46.717) | <.001 |
|  | Age^-1 | -405.474 (-481.464 to -329.485) | <.001 |
|  | Age^2 | -0.004 (-0.005 to -0.004) | <.001 |
|  | Age^-1 * lowest quintile | -24.421 (-108.596 to 59.755) | .570 |
|  | Age^2 * lowest quintile | 3.46e-3 (8.21e-3 to1.29e+3) | .153 |
